# Failure to detect *M. avium* subspecies *paratuberculosis* in Johne’s disease using a proprietary fluorescent in situ hybridization assay

**DOI:** 10.1186/s13104-018-3601-5

**Published:** 2018-07-21

**Authors:** Robert J. Greenstein, Liya Su, Peter S. Fam, Judy R. Stabel, Sheldon T. Brown

**Affiliations:** 1 Department of Surgery, James J. Peters Veterans Affairs Medical Center, Bronx, NY USA; 2 Laboratory of Molecular Surgical Research, James J. Peters Veterans Affairs Medical Center, Bronx, NY USA; 3 Mental Illness Research Education and Clinical Center (MIRECC), James J. Peters Veterans Affairs Medical Center, Bronx, NY USA; 4 Johne’s Disease Research Project, USDA-ARS, National Animal Disease Center, Ames, IA 50010 USA; 5 Infectious Disease Section, James J. Peters Veterans Affairs Medical Center, Bronx, NY USA; 6 Department of Medicine, Icahn School of Medicine at Mt. Sinai, New York, NY USA

**Keywords:** In situ hybridization, *Mycobacterium avium* subspecies *paratuberculosis*, Mycobacteria, Johne disease

## Abstract

**Objectives:**

*Mycobacterium avium* subspecies *paratuberculosis* (MAP) causes Johne’s disease in ruminants. The “gold standard” of MAP detection is by culture, DNA sequencing possibly supplemented by identification of Ziehl–Neelsen positive mycobacteria. The purpose of this study was to evaluate a proprietary (Affymetrix™ RNA view^®^) fluorescent in situ hybridization (FISH) assay for MAP RNA. Intestine from a steer with documented Johne’s disease was assayed according to the manufacturer’s instructions. Probes were custom designed for MAP and bovine β-actin (as the eukaryotic housekeeping gene) from published genomes. We attempt to prevent false positive signal in the “no-probe” control, by modifying wash solutions, using recommended hydrochloric acid titration and different fluorescent filters (TritC for Texas Red and “Hope” for Cy-5).

**Results:**

Repetitively, false positive signal was observed in our “no probe” negative control. Attempts to correct this according to the manufacturers suggestions, and with multiple derivative techniques have been unsuccessful. It is concluded that when performed according to manufactures instruction and with multiple variations on the manufactures recommended suggestions to correct for false positive signal, that the Affymetrix™ RNA view^®^ cannot be used to detect MAP in pre-frozen intestine of cattle with Johne’s disease.

**Electronic supplementary material:**

The online version of this article (10.1186/s13104-018-3601-5) contains supplementary material, which is available to authorized users.

## Introduction

*Mycobacterium avium* subspecies *paratuberculosis* (MAP) causes Johne’s disease in both agricultural and wild animals, at considerable cost and animal morbidity and mortality [1]. The gold standard of diagnosis of Johne’s disease is culture of MAP [2]. In animals with Johne’s disease, this is a reliable, but time-consuming process. Multiple other diagnostic modalities exist for detecting mycobacteria in general [2–5] and MAP in particular [6–10]. Following the detection of putative MAP, confirmation usually requires the identification of the DNA sequence IS900 which is unique to MAP [11]. Detection usually requires that MAP have a cell wall [7, 12].

There is the possibility that MAP may be zoonotic [13–16]. However, in humans MAP exists in the cell wall deficient form. Although MAP has been cultured from humans with Crohn’s disease [17], this is difficult, few laboratories can do so [18–22], and up to 18 months may be required for the organism to reconstitute its cell wall [17]. The detection of MAP DNA does not signify that the organism was viable [23]. In contrast, detecting MAP RNA implies viability [16]. It would therefore be of use to develop an assay that reliably and rapidly identifies MAP RNA in possibly infected intestine.

We herein report on our attempts to develop a fluorescent in situ hybridization (FISH) assay of MAP RNA, using a proprietary RNA amplification technique (Affymetrix™ RNA view^®^). The tested tissue was previously frozen intestine of cows with known Johne’s disease.

## Main text

This study was approved by the Research & Development Committee at the VAMC Bronx NY (0720-06-038).

Bovine ileal intestinal tissue were from two sources. A steer with Johne’s disease (a gift of Robert Whitlock DVM PhD. University of Pennsylvania School of Veterinary Medicine. Bounders Green. PA. USA) was stored at − 80 °C until processed. Multiple samples were obtained from Judy Stabel Lead Scientist, Johne’s Disease Research Project USDA Ames Iowa. Serial 12 µ sections were cut at − 20 °C (Leica CM 3050S microtome; Cryostar Industries). Sequential sections were placed on Affymetrix recommended slides (Superfrost Plus microscope slides # 12-550-15 Fisher Industries), vacuum packed and stored at − 80 °C until processed for an experiment.

Affymetrix ViewRNA ISH tissue 2-Plex Assay^®^ (Affymetrix: ThermoFisher USA). Affymetrix generated probes using the published sequence for MAP [11]. (Affymetrix name: *M. tuberculosis* Is900: Cat # VF1 19496: Lot # 195634523: Probe type 1). For bovine β-actin (Bos Taurus actb: NCBI Reference Sequence: NM_173979.3 (Affymetrix name: Bos Taurus Actb: Cat# VF6 20062: Lot # 200642784: Probe Type 6). These probes are proprietary to Affymetrix.

The assay was carried out according to the published ViewRNA™ ISH Tissue 2-Plex Assay instructions (Protocol Guide for RNA in situ Hybridization. Affymetrix: ThermoFisher USA). Hybridization was performed using a Slide Moat^®^ Model 2400 (Boekel Scientific) Accuracy of temperature in the multiple steps were confirmed using a suggested thermocouple (ViewRNA™ Temperature Validation Kit Cat. # QV0523 ThermoFisher).

**Fig. 1:**
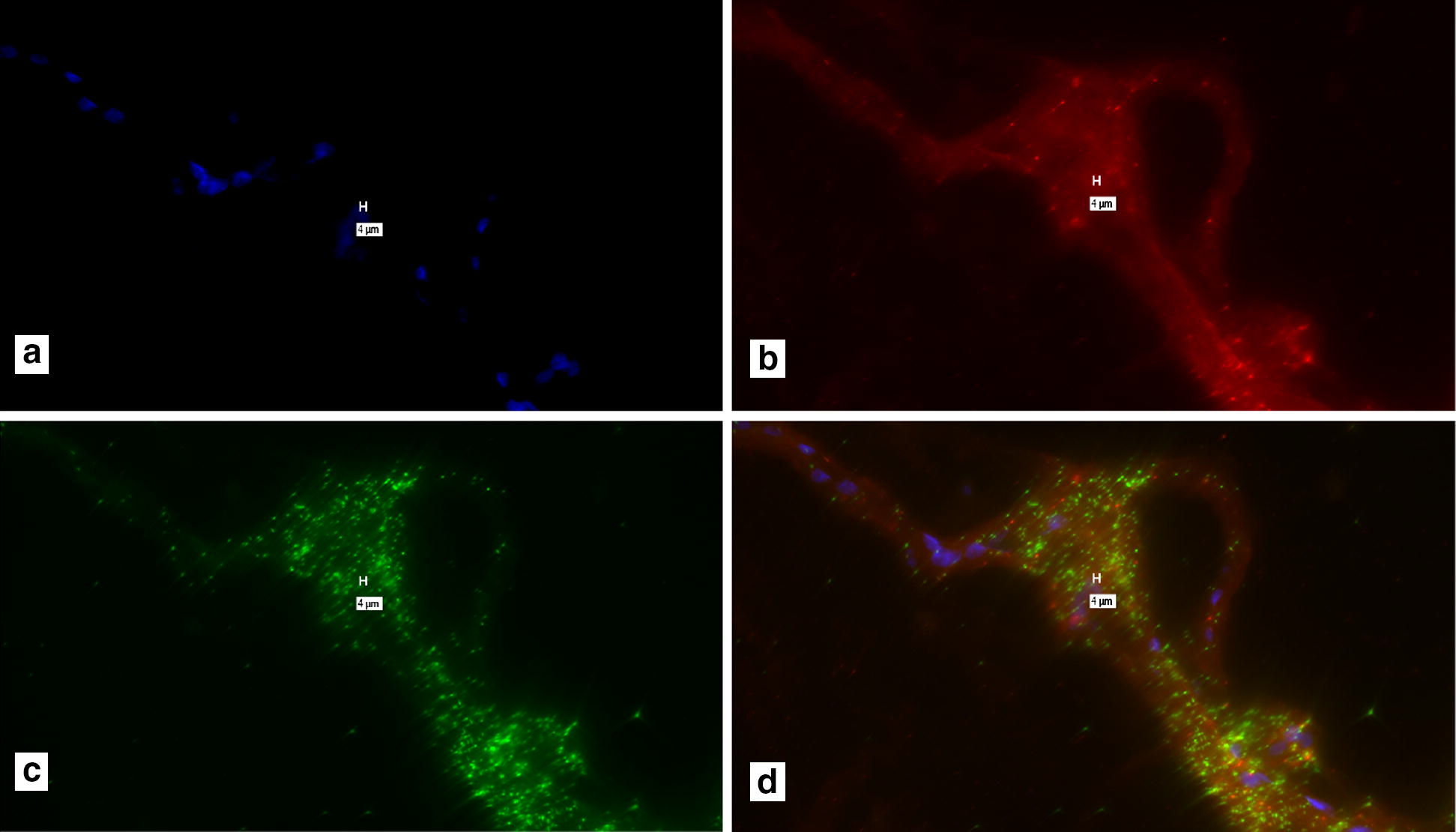
A composite of four images of the same section, of Bovine Johne’s disease tissue. **a** DAPI; **b** Texas Red (IS900); **c** Cy-5 (Bovine β-actin) **d** composite of **a**–**c**. With probes: Note “positive: signal in **b**–**d**. Marker bars, in µm, indicate magnification of x 40

**Fig. 2:**
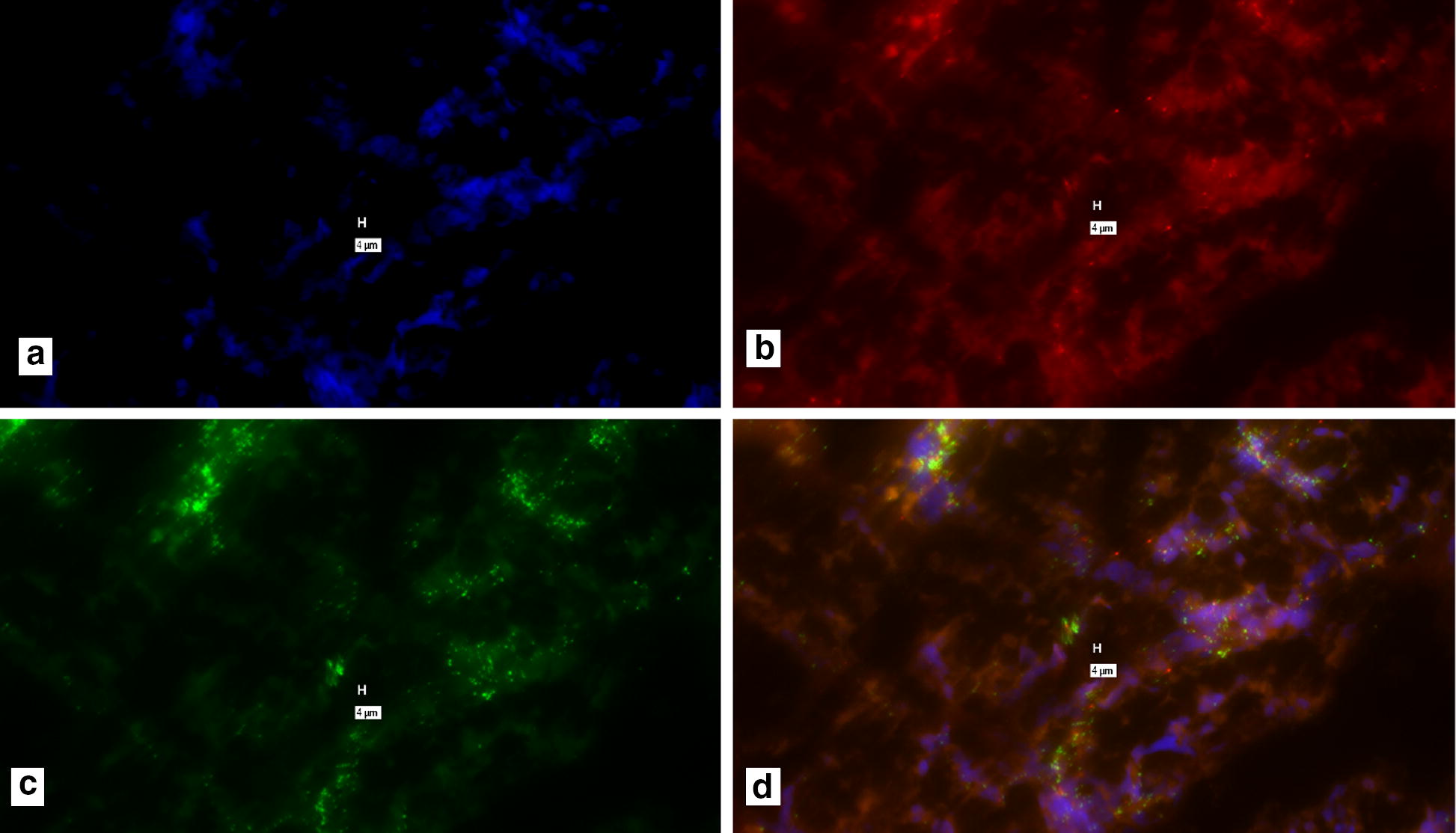
“No-probe” control for Fig. 1. Processed identically as in Fig. 1, during the same experiment, but no probes were added during the hybridization step. **a** DAPI; **b** Texas Red (IS900); **c** Cy-5 (Bovine β-actin) **d** composite of **a**–**c**. No- probes: Note “positive” signal in the “No-probe” control **b**–**d**. Marker bars, in µm, indicate magnification of x 40

A clear background in the control slide from which probes had been excluded during the Probe Set Hybridization steps could not be obtained repetitively (Figs. 1 and 2). The hypothesis that contamination of the “no-probe” control slide, occurred during washing was tested. First compared washing both slides with the same wash solution (Additional files 1 and 2). Next, different wash solutions were compared (Additional files 3 and 4).

**Fig. 3:**
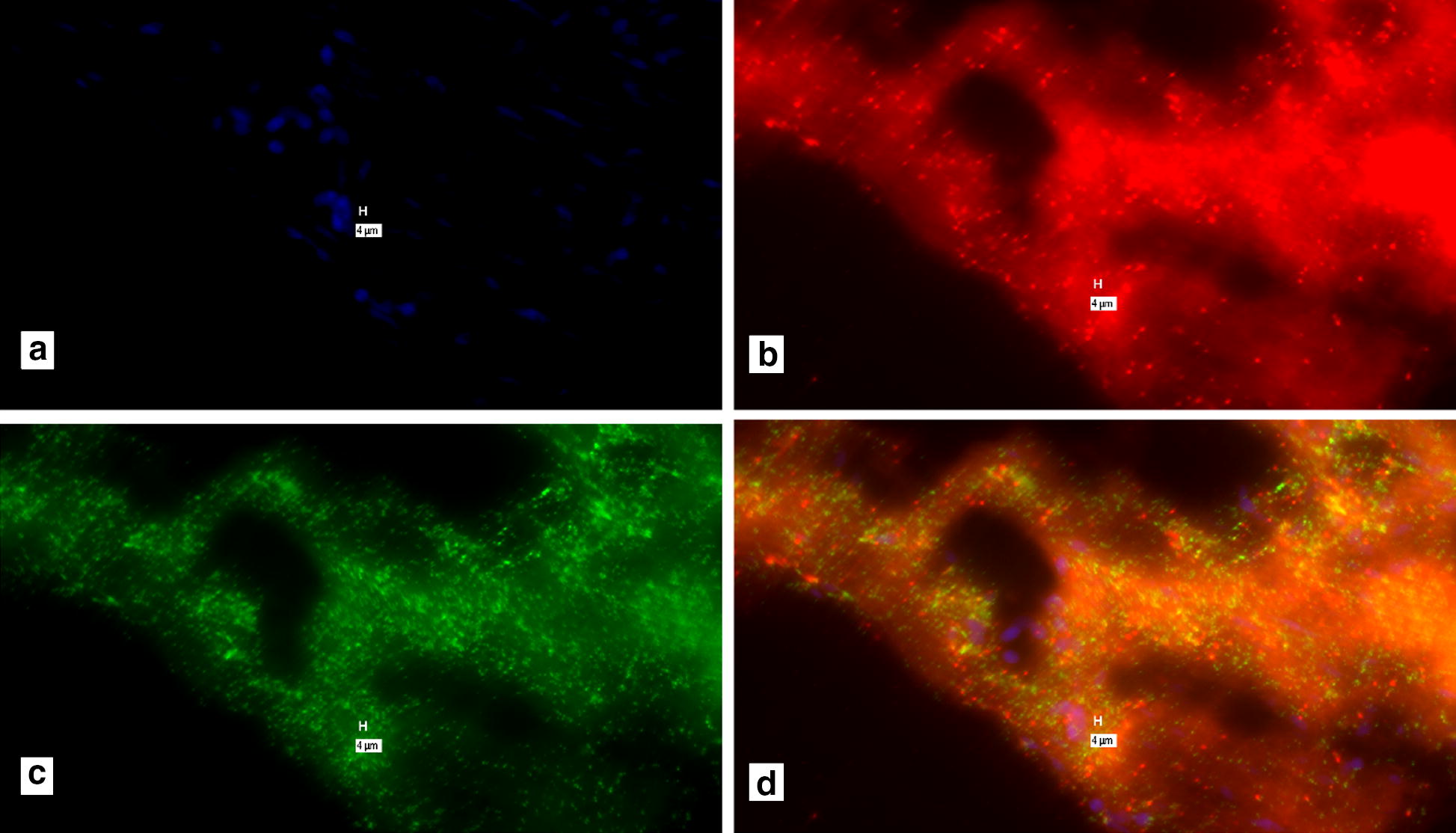
“No-probe” negative control for Additional file 7. This was a 35-min exposure to 0.2 M HCl. **a** DAPI, identifying the presence of DNA. Note the positive signal in this “No-probe” control for **b**–**d**. Marker bars, in µm, indicate magnification of x 40

The Affymetrix ViewRNA™ ISH Tissue 2-Plex Assay instructions, suggests that pretreating with HCl obviates false positive signal (Affymetrix: Protocol Guide for RNA in situ Hybridization. Troubleshooting for high background. Page 71). Initially, the manufacturer recommended 0.2 M HCl for 10 min (Additional file 5), was compared with the “No-Probe” control (Additional file 6). Because of ongoing signal in the no-probe control we performed a time titration using 0.2 M HCl for 25 and 35 min. Additional file 9 presents data for the 35 min 0.2 M HCl exposure “with-probe” and Fig. 3, 35 min 0.2 M HCl exposure of the “No-Probe” control). Increasing the concentration of HCl from 0.2 to 0.4 M HCl to 0.6 M HCl was evaluated (Additional files 8 and 9). Because of the possibility of neutralization of the HCl, 400 µl 0.2 M HCl was added, removed immediately and then added an additional 400 µl added for the reported time.

Slides were read using a Keyence BZ-X710 Fluorescence microscope with a BZ-X700E controller. Filters used were OP-87762 for DAPI (Excitation 340–380 nm; Emission 435–485 nm), OP87763 for GFP (Excitation 480–490 nm; Emission 500–550 nm), OP87764 for TritC (Excitation 530 ± 20 nm Emission 590 ± 20 nm; Dichroic 562 nm), OP87765 for Texas-Red (Excitation 540–580; Emission 592–667.5 nm), OP87766 for Cy-5 (Excitation 590–650 nm; Emission 662.5–667.5 nm) and a blank OP87767 filter cube was to hold the custom filter for “Hope” (All from Keyence USA: Elmwood Park. NJ. USA). Custom filters were used for “Hope” (Excitation 630 ± 20 nm; Emission 775 ± 25 nm; Dichroic 750 nm) (#49019-UF1Keyence BZX Un-Mounted ET Cy5 Longpass: Chroma Technology Corp; Bellows Falls. VT).

Images were compared for filters with different Absorption and Excitation spectra (Additional files 10 and 11). For Texas Red, the comparison was with TritC. For Cy-5 the comparison was with “Hope.”

To evaluate whether false positive signal could be ascribed to artifact from the Keyence BZ-X710, probe and No-Probe negative control slides were evaluated on an alternative microscope (Additional file 12. Panoramic 2503D. HisTech^®^; Budapest, Hungary) as well as a Zeiss ApoTome Imager 1.

Multiple bovine intestinal specimens were obtained from the Johne’s Disease Research Project USDA Ames, Iowa. These included both healthy controls as well as samples from animals with Johne disease. (Additional file 13).

During our efforts to obviate false positive signal we repetitively contacted the Technical staff at ThermoFisher Affymetrix provided us with a Rat Kidney Control Kit that contained three slides. Two had reciprocal probe sets (types 1 and 6). The third slide was ThermoFisher’s “No Probe” control. The “No-probe” control images of Rat Kidney are presented in Additional file 14.

Purportedly positive signal for both IS900/MAP and Bovine β-actin, our house-keeping control gene (Fig. 1). However, the control slide, where no probes were added during the hybridization step, shows similar “positive” signal (Fig. 2). These indicate that false positive signal is seen with recommended hybridization conditions.

To determine whether there was contamination of the negative control slide by probes during the post hybridization wash, washing the slides in the same solution both with probes were compared (Additional file 1) and without probes (Additional file 2). False positive signal is seen in the negative Control slide (Additional file 2).

Using separate wash solution for the slide hybridized with probes (Additional file 3) and separate wash solution for the no-probe slide were compared (Additional file 4). Again, false positive signal is seen in the negative control slide.

Next slides were pretreated in 0.2 M HCl for 15 min, prior to hybridization with probes (Additional file 5) and without probes (Additional file 6). Again, false positive signal is seen in the negative control slide (Additional file 6).

The period of exposure of 0.2 M HCl was prolonged to 25 and 35 min. “Positive” signal is seen in both the 35-min 0.2 M HCl exposure slides with probes (Additional file 7) and the negative control, without probes (Fig. 3).

The concentration of the HCl was increased to 0.4 and 0.6 M HCl for 15 min. “Positive” signal is seen in the 0.6 M HCl exposure for both the slide with probes (Additional file 8) and the negative control, without probes (Additional file 9).

At the recommendation of the technical staff at Affymetrix different filters were compared. For IS900/MAP we compared Texas Red and TritC. For Bovine β-actin we compared Cy-5 with “Hope” (see “Main text” section). Although there is a difference in intensity there is no difference in the “positive” signal seen in the slide with probes (Additional file 10) and the negative “No probe” control (Additional file 11).

An alternative HisTech^®^ combined fluoroscopic/Brightfield imager was used (see “Main text” section). Again “positive” signa is observed, in the “No-Probe” negative control (Additional file 12).

A “No-Probe” representative result on the intestinal tissue sample from the Johne’s Disease Research Project USDA Ames Iowa is shown in Additional file 13. False positive signal is most noticeable in the Cy5 image (Additional file 13c).

In Additional file 14 “No-Probe” false positive signal is seen in Rat Kidney Control kit slides prepared by the technical staff at Affymetrix. The only role of our laboratory was to read the slides. In the images presented the filters were Cy5 and Texas Red.

A proprietary FISH assay has been performed according to the recommended conditions of the vendor. Purportedly positive signal was detected for both MAP (IS900) and our eukaryotic housekeeping gene, bovine β-actin. However, repetitively the “No-Probe” negative control for a given experiment showed obviously false “positive” signal.

A multitude of experimental perturbations have been performed, in attempts to get appropriate negative controls when No-Probes were used in control slides. These included evaluating whether inadvertent probe contamination occurred during the washing process, following Target Probe Set Hybridization. False positive signal was observed irrespective of whether the same or completely different wash solutions were used. It is concluded that the “positive” signal in “No-Probe” negative controls cannot be ascribed to probe contamination during the post-hybridization wash.

Affymetrix recommends pre-treating with 0.2 M HCl for 15 min to prevent false positive signal. As this was ineffective, the time of 0.2 M HCl exposure has been extended, and the concentration of HCl increased to a maximum of 0.6 M. None of these HCl perturbations obviated “positive” signal in “No-Probe” negative controls. It is concluded that the use of acid to prevent “positive” signal in “no-probe” negative controls is of no use when studying bovine small intestine using the Affymetrix ViewRNA™ ISH Tissue 2-Plex Assay.

Following consultation with the technical staff at Affymetrix, alternative filterswere employed. For Texas-Red, TritC was substituted. For Cy-5 a custom recommended filter set “Hope” was used (see “Main text” section). Although the comparison showed different intensity (Additional files 10 and 11), both sets of filers show the same “positive” signal in both the probe samples and the “No-Probe” controls. It is concluded that the use of the manufacturer recommended different filters to prevent “positive” signal in “no-probe” negative controls is of no use when studying bovine small intestine using the Affymetrix ViewRNA™ ISH Tissue 2-Plex Assay.

Similar positive signal in the “No-Probe” control was obtained with recently received samples from Johne’s Disease Research Project USDA Ames Iowa. Likewise, positive signal was found in “no-probe” controls of Rat Kidney that had been processed at Affymetrix. The only action taken was to read these Affymetrix slides on two microscopes available to our laboratory.

Finally, the “positive” signal observed with the Keyence microscope is found with the alternative HisTech imager as well as the Zeiss Apotome Imager Z1.

It is concluded that when the assay is performed according to the Affymetrix ViewRNA™ ISH Tissue 2-Plex Assay recommended instructions, it cannot be used for FISH studies to identify the RNA of MAP on previously frozen Johne’s disease, bovine intestine.

## Limitations

When a change in gene expression is being quantified, a low FISH signal to noise background may be acceptable. However, in this study we asked a binary question. Is MAP present or absent in a given sample? Especially when the target is expected to be in low abundance, any background may result in a false positive interpretation and is unacceptable. Thus, these conclusions apply only to frozen intestinal tissue where we are attempting to identify MAP and not to other scientific investigations.

The ViewRNA™ ISH Tissue 2-Plex Assay is designed to study tissue, not isolated cells. Accordingly, pure bacterial cultures cannot be used as controls. Although the ThermoFisher provided No-Probe slides had positive signal, it was not stated whether these had been frozen prior to processing. These studies were not performed on fresh tissue. Recently harvested bovine samples were evaluated, nevertheless they had been pre-frozen and shipped on dry-ice at − 20 °C. Therefore, these comments should only be applied to frozen, not fresh, intestine.

## Additional files

**Additional file 1.** Following hybridization, slides with and without probes, were washed together. With probes: Note “positive” signal in Additional file 1: b, c and d.

**Additional file 2.** “No-probe” negative control for Additional file 1. Processed in the same experiment. The same wash solution was used for the slides in Additional files 1 & 2. No- probes: Note the “positive” signal in the “No-probe” control in Additional file 2: b, c & d.

**Additional file 3.** Comparison of different washing solutions. Slides visualized in Additional file 3 & 4 were processed identically, in the same experiment, but were washed using separate wash solutions. With probes: Note “positive” signal in Additional file 3: b, c and d.

**Additional file 4.** “No-probe” negative control for Additional file 3. No- probes: Note the “positive” signal in the “No-probe” control Additional file 4: b, c and d.

**Additional file 5.** Comparison of pretreating with Hydrochloric acid (0.2 M/15 minutes recommended by Affymetrix®). With probes: Note “positive” signal in Additional file 5: b, c and d.

**Additional file 6.** “No-probe” negative control for Additional file 5. The slide was processed identically as in Additional file 5, bathed in 0.2M HCl. No- probes: Note the “positive” signal in the “No-probe” control Additional file 6: b, c and d.

**Additional file 7.** Comparison of longer exposure (35 minutes) of 0.2 M HCL. With probes: Note “positive” signal in Additional file 7: b, c and d. Compare these with the “”No probe” controls presented in Figure 3 that also show “positive” signals in Figures 3: b, c & d.

**Additional file 8.** Comparison of increased concentration (0.6M) HCl. Slide exposed for 20 minutes to HCL and probes. With probes: Note “positive” signal in Additional file 8 b, c and d.

**Additional file 9.** “No-probe” negative, 0.6M HCl control for Additional file 9. No- probes: Note the “positive” signal in the “No-probe” control Additional file 9: b, c and d.

**Additional file 10.** Comparison of different fluorescent filters (See “Main text”.) “Positive” probe control. For IS 900 MAP Upper left (a) is Texas Red. Upper right (b) is TritC. For Bovine β-actin, (c) is Cy-5 and (d) is “Hope.” Identical section of slide. Although there is a slight difference in intensity, purportedly “positive” signal is seen with both sets of filters, when (a) is compared with (b), as well as when (c) is compared with (d).

**Additional file 11.** “No-probe” negative control for Additional file 10. Slide processed identically to that in Additional file 10. Upper left (a) is Texas Red. Upper right (b) is TritC. Bottom left (c) is Cy-5 and bottom right (d) is “Hope.” Identical section of slide. Note the positive signal in this “No-probe” control. As in Additional file 10, without probes although there is a slight difference in intensity, “positive” signal is seen with both sets of filters.

**Additional file 12.** A comparison, using an alternative imager (HisTech®. See “Main text” section for details.) With (a & b) & without probes (c & d). Brightfield are a & c. Composite fluorescent of DAPI, Cy-5 & Texas-Red are b & d. “Positive” fluorescent signal is seen for both CY5 and Texas-Red in both b & d. Note difference in magnification between Brightfield and Fluorescent images.

**Additional file 13.** A “No-probe” control on a specimen from Ames Iowa visualized with Bright Field (Additional file 13 a) Texas Red (Additional file 13 b) and Cy-5 (Additional file 13 c) Composite of DAPI, Texas Red & Cy5 (Additional file 13 d.) Note the positive signal in this “No-probe” control.

**Additional file 14.** Affymetrix supplied Rat Kidney “No-probe” control slide. “a” = Bright field x100. “b” = Composite of DAPI, Texas Red & Cy-5. “c” = Composite of DAPI, TritC and “Hope”. Note the positive signal in the “No-probe” control.

## Abbreviations

MAP: *M. avium* subspecies *paratuberculosis*
FISH: fluorescent in situ hybridization
HCl: hydrochloric acid
