# Supplementary figures and images for: Failure to detect M. avium subspecies paratuberculosis in Johne’s disease using a proprietary fluorescent in situ hybridization assay
Source: BMC Res Notes. 2018 Jul 21;11:498. doi: 10.1186/s13104-018-3601-5 (PMC6054717; doi:10.1186/s13104-018-3601-5)

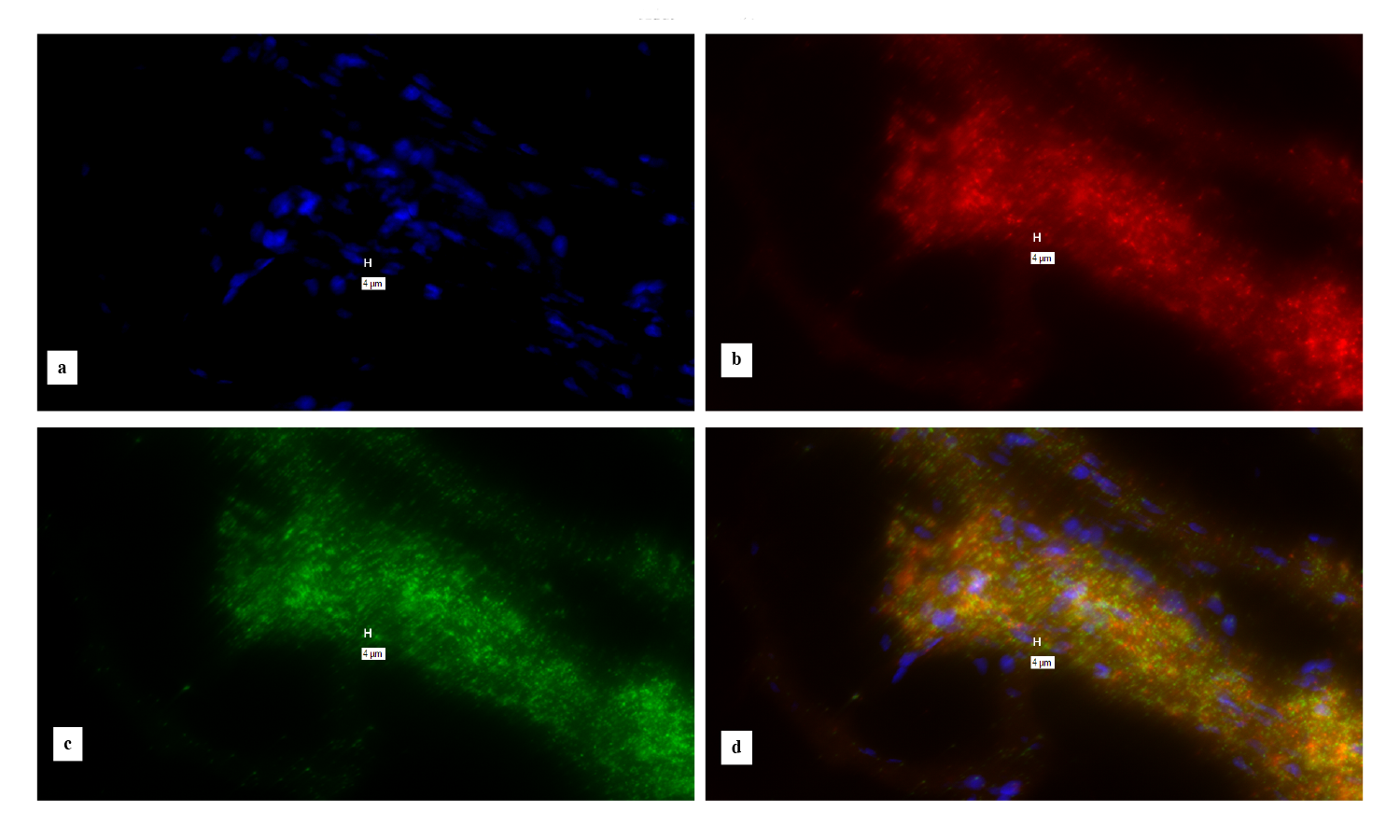

Supplement: Supplementary file 1 — Additional file 1. Following hybridization, slides with and without probes, were washed together. With probes: Note “positive” signal in Additional file 1: b, c and d. [file 13104_2018_3601_MOESM1_ESM.tif]

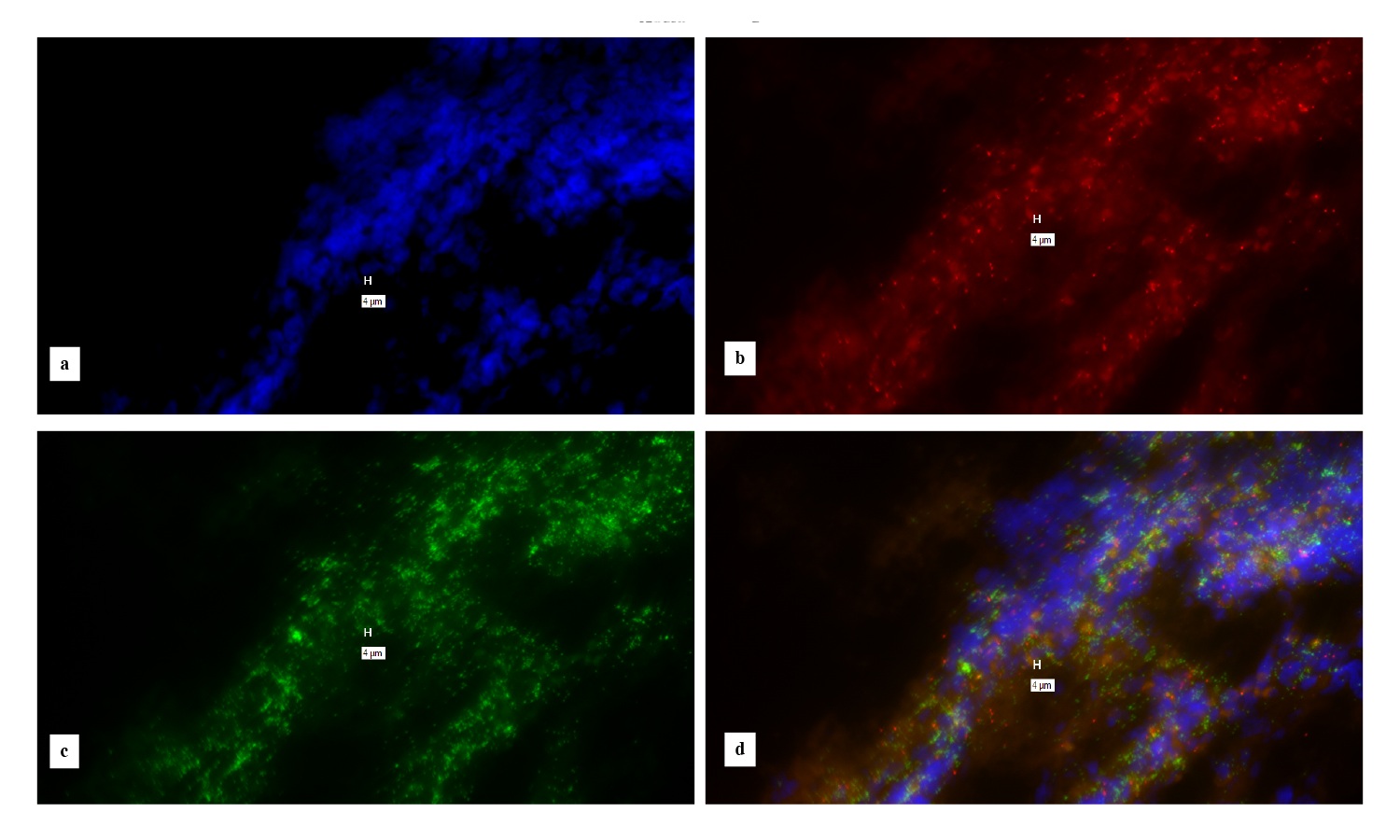

Supplement: Supplementary file 2 — Additional file 2. “No-probe” negative control for Additional file 1. Processed in the same experiment. The same wash solution was used for the slides in Additional files 1 & 2. No- probes: Note the “positive” signal in the “No-probe” control in Additional file 2: b, c & d. [file 13104_2018_3601_MOESM2_ESM.tif]

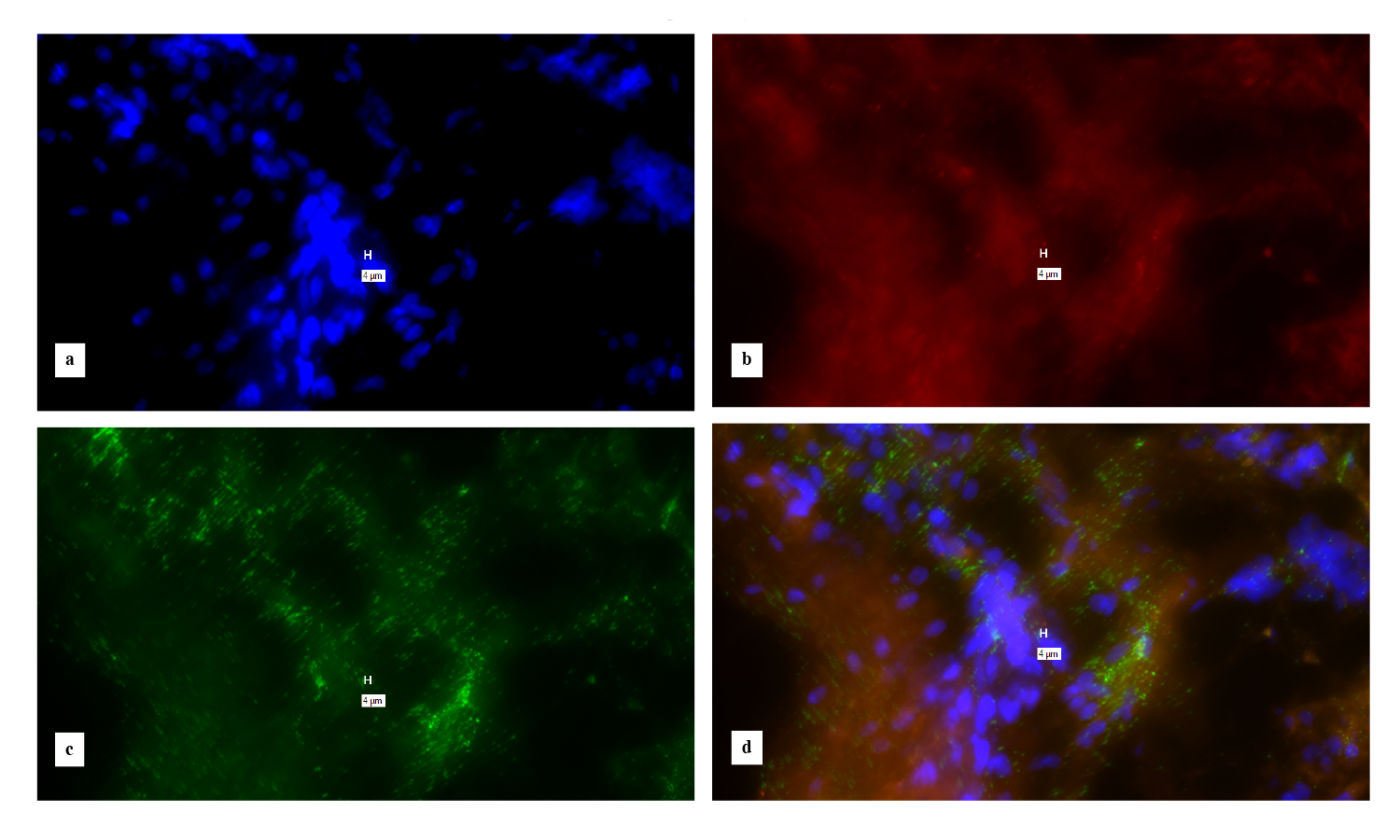

Supplement: Supplementary file 3 — Additional file 3. Comparison of different washing solutions. Slides visualized in Additional file 3 & 4 were processed identically, in the same experiment, but were washed using separate wash solutions. With probes: Note “positive” signal in Additional file 3: b, c and d. [file 13104_2018_3601_MOESM3_ESM.tif]

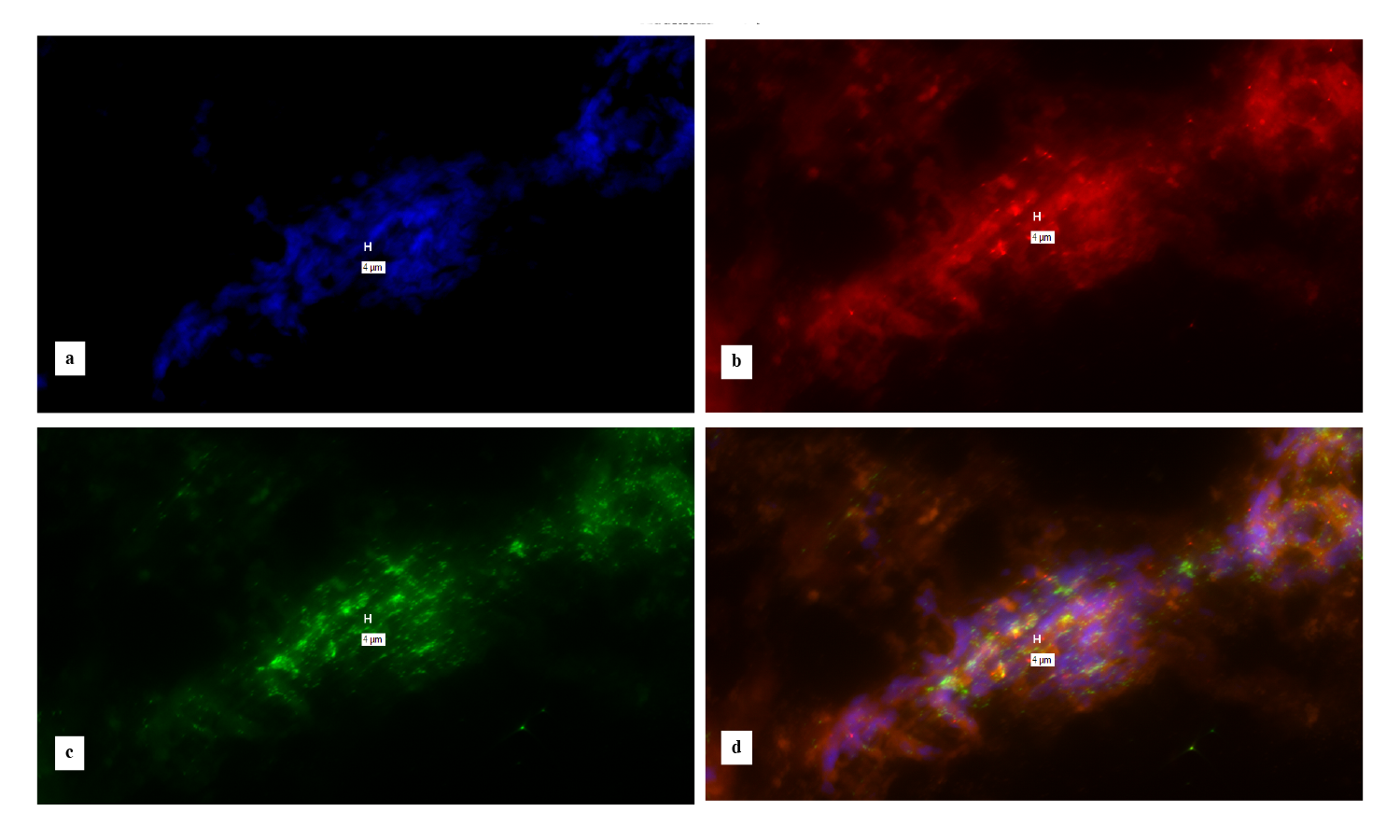

Supplement: Supplementary file 4 — Additional file 4. “No-probe” negative control for Additional file 3. No- probes: Note the “positive” signal in the “No-probe” control Additional file 4: b, c and d. [file 13104_2018_3601_MOESM4_ESM.tif]

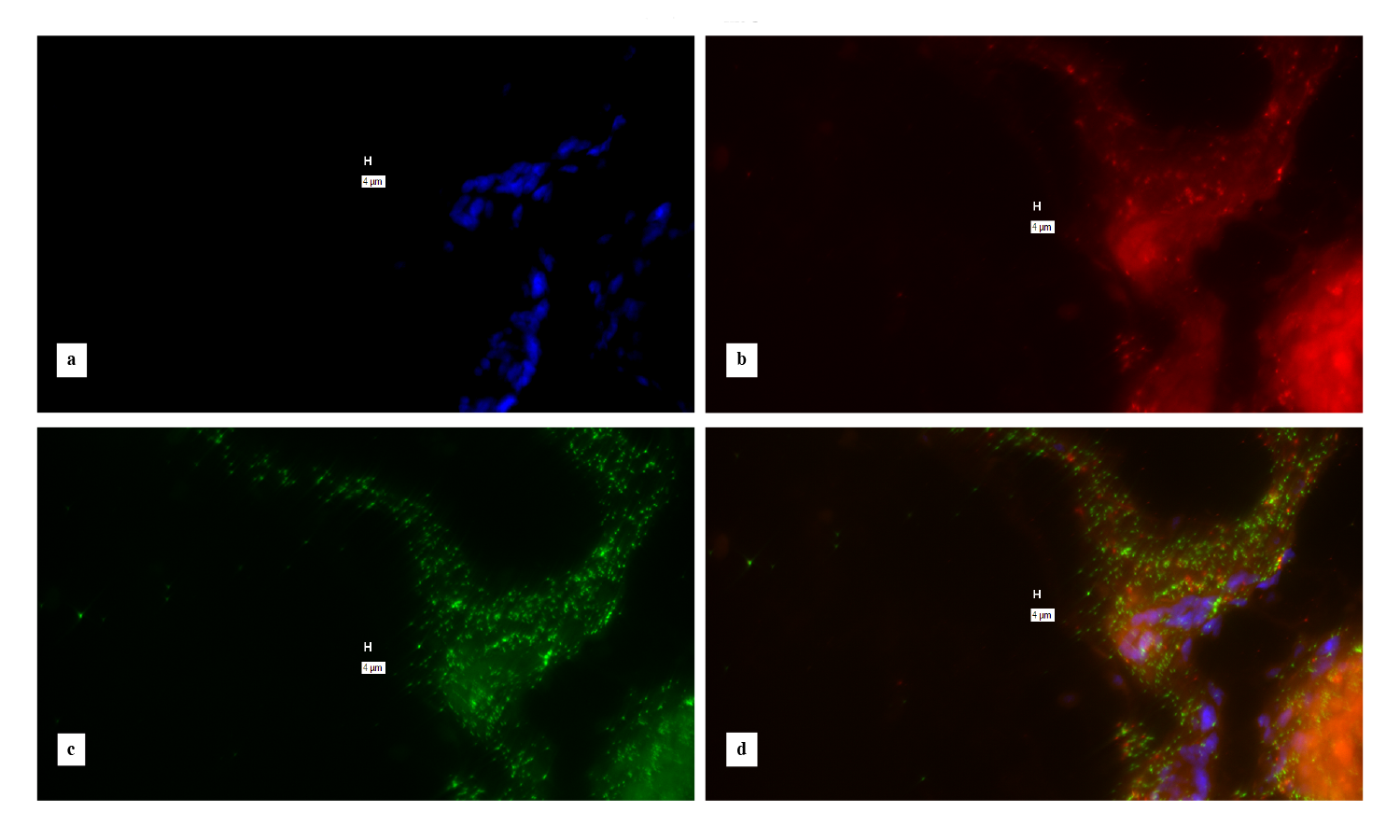

Supplement: Supplementary file 5 — Additional file 5. Comparison of pretreating with Hydrochloric acid (0.2 M/15 minutes recommended by Affymetrix®). With probes: Note “positive” signal in Additional file 5: b, c and d. [file 13104_2018_3601_MOESM5_ESM.tif]

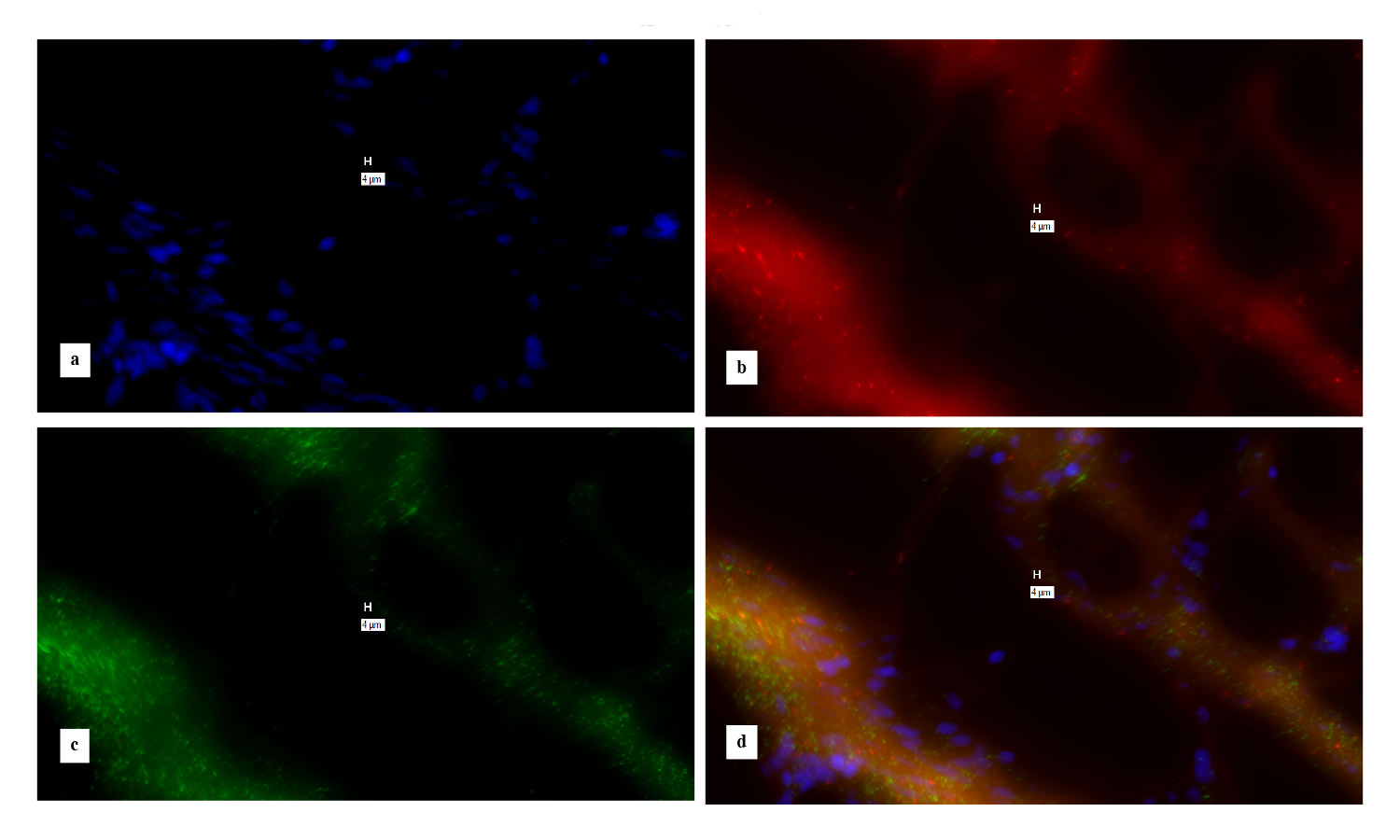

Supplement: Supplementary file 6 — Additional file 6. “No-probe” negative control for Additional file 5. The slide was processed identically as in Additional file 5, bathed in 0.2M HCl. No- probes: Note the “positive” signal in the “No-probe” control Additional file 6: b, c and d. [file 13104_2018_3601_MOESM6_ESM.tif]

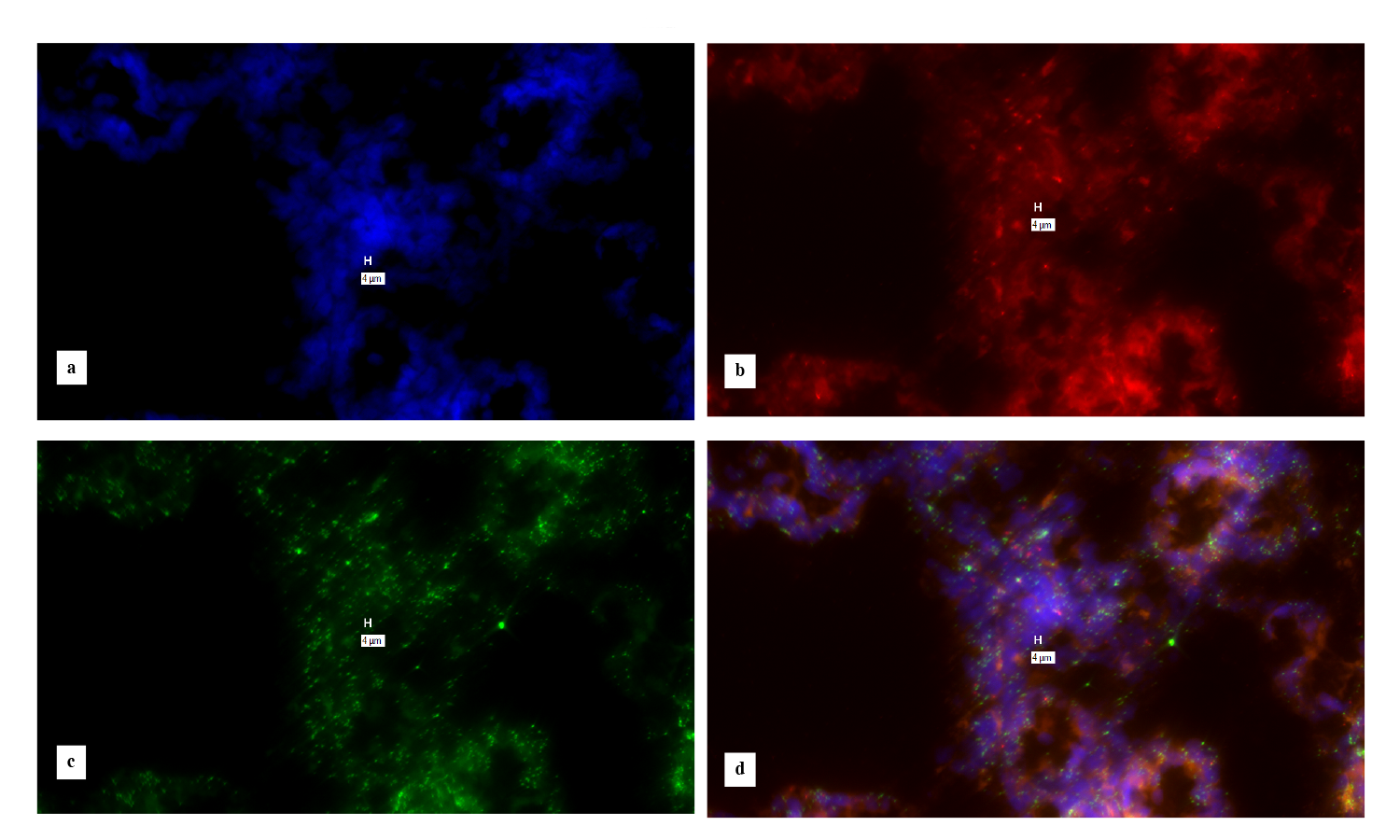

Supplement: Supplementary file 7 — Additional file 7. Comparison of longer exposure (35 minutes) of 0.2 M HCL. With probes: Note “positive” signal in Additional file 7: b, c and d. Compare these with the “”No probe” controls presented in Figure 3 that also show “positive” signals in Figures 3: b, c & d. [file 13104_2018_3601_MOESM7_ESM.tif]

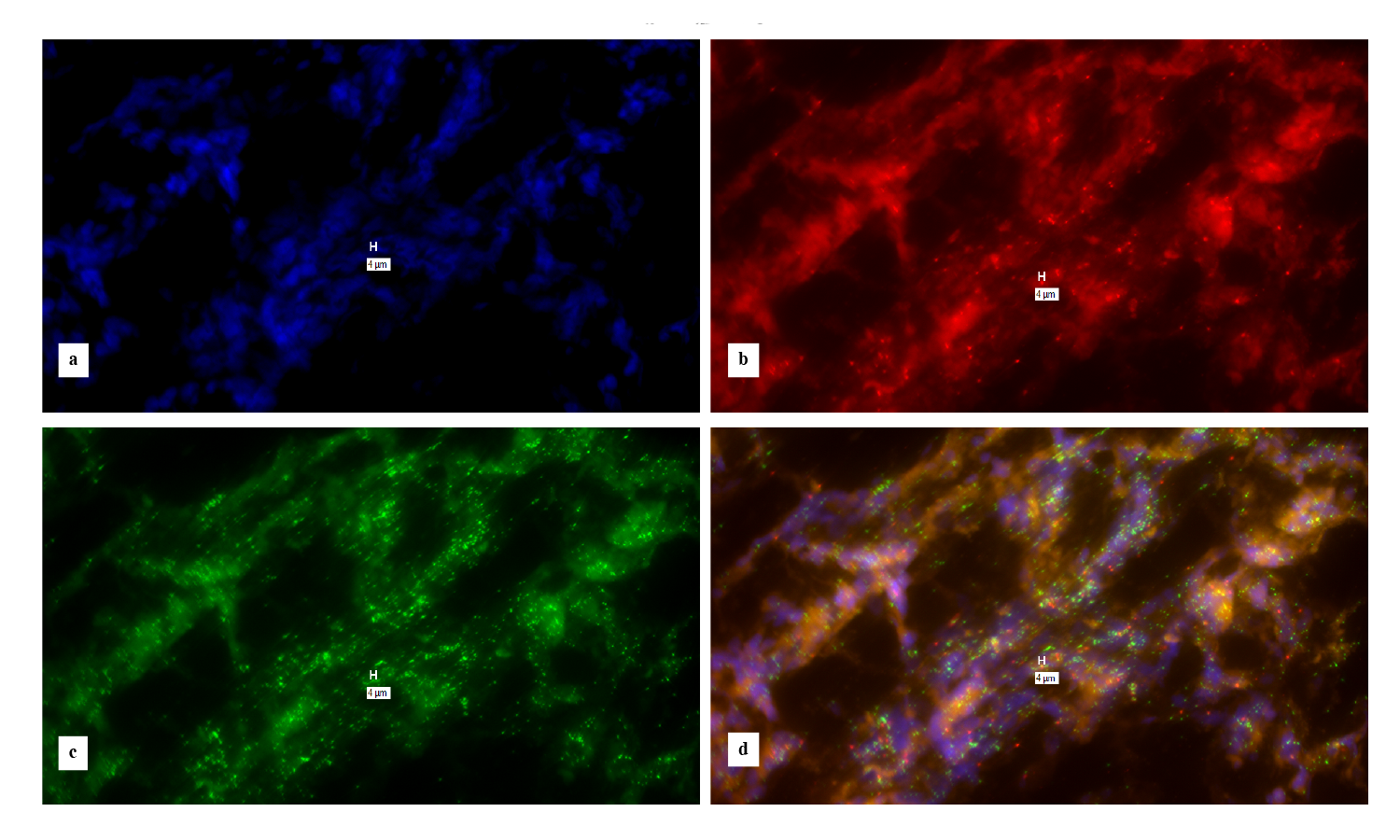

Supplement: Supplementary file 8 — Additional file 8. Comparison of increased concentration (0.6M) HCl. Slide exposed for 20 minutes to HCL and probes. With probes: Note “positive” signal in Additional file 8 b, c and d. [file 13104_2018_3601_MOESM8_ESM.tif]

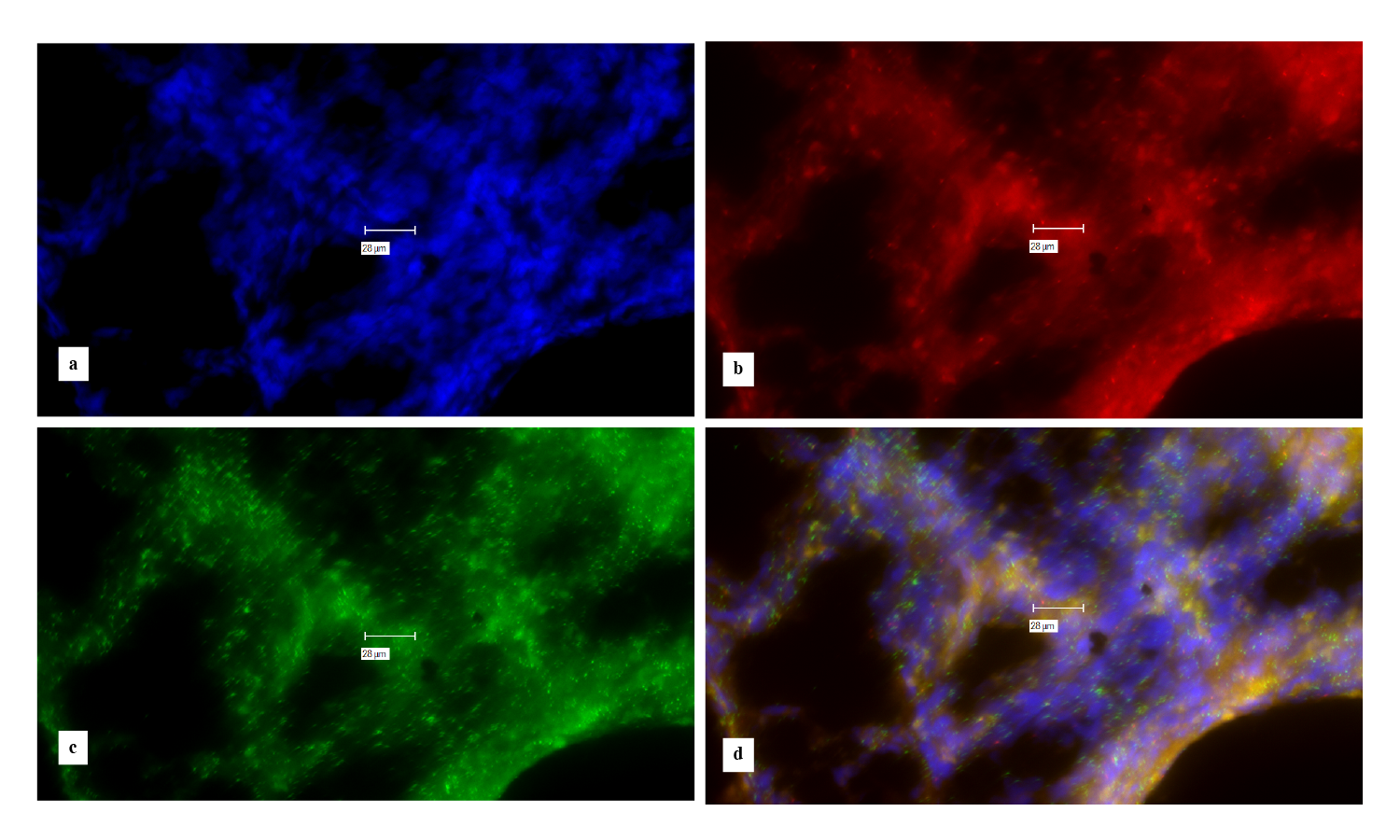

Supplement: Supplementary file 9 — Additional file 9. “No-probe” negative, 0.6M HCl control for Additional file 9. No- probes: Note the “positive” signal in the “No-probe” control Additional file 9: b, c and d. [file 13104_2018_3601_MOESM9_ESM.tif]

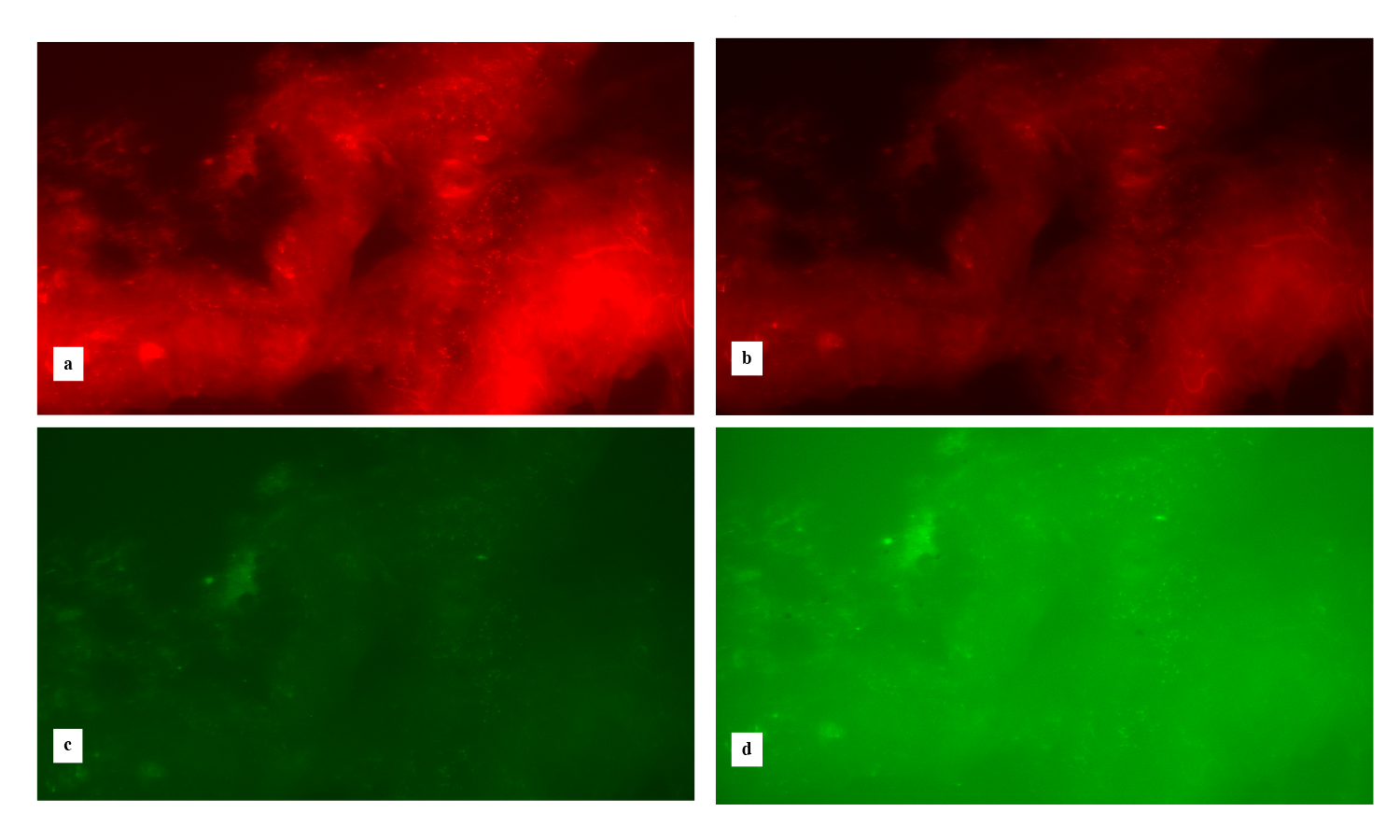

Supplement: Supplementary file 10 — Additional file 10. Comparison of different fluorescent filters (See “Main text”.) “Positive” probe control. For IS 900 MAP Upper left (a) is Texas Red. Upper right (b) is TritC. For Bovine β-actin, (c) is Cy-5 and (d) is “Hope.” Identical section of slide. Although there is a slight difference in intensity, purportedly “positive” signal is seen with both sets of filters, when (a) is compared with (b), as well as when (c) is compared with (d). [file 13104_2018_3601_MOESM10_ESM.tif]

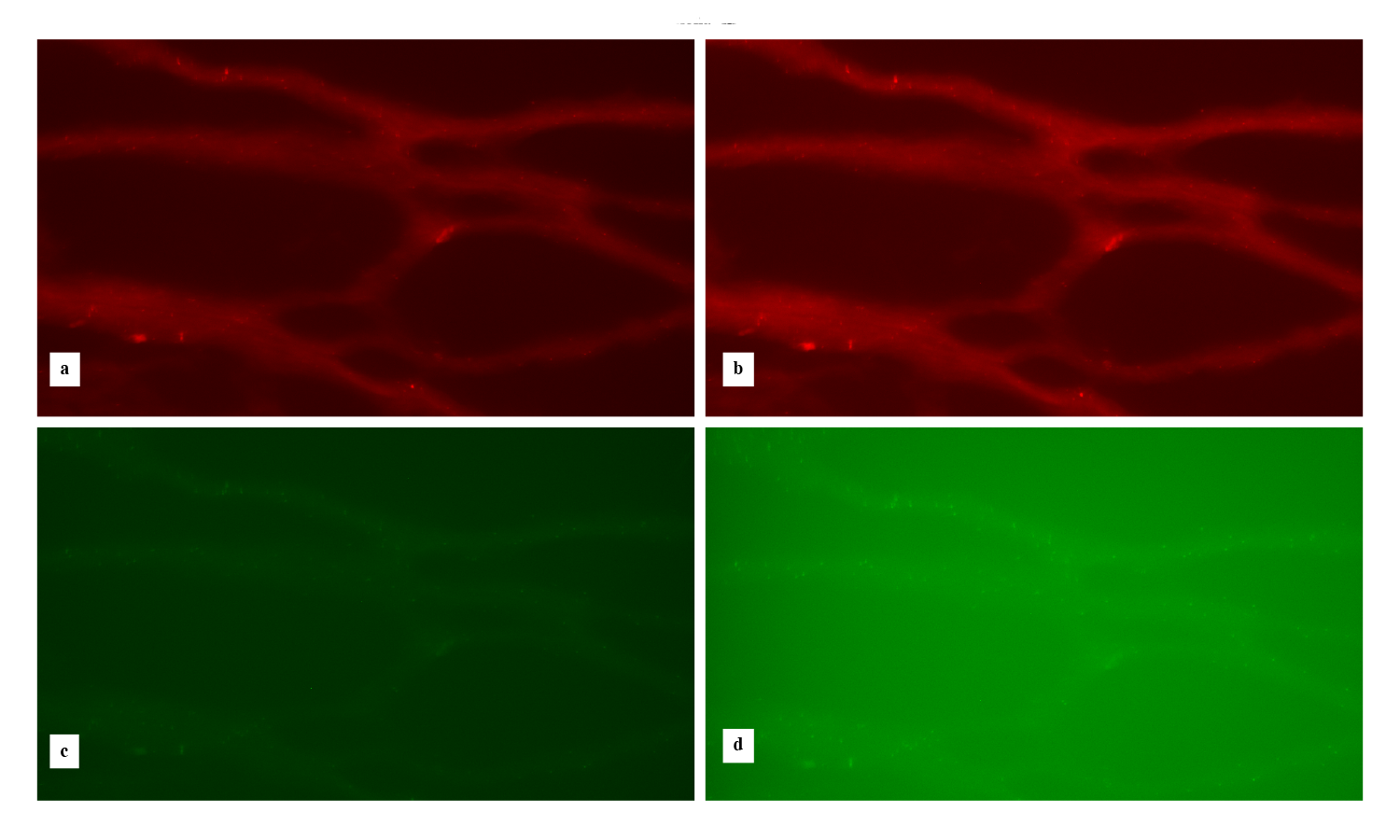

Supplement: Supplementary file 11 — Additional file 11. “No-probe” negative control for Additional file 10. Slide processed identically to that in Additional file 10. Upper left (a) is Texas Red. Upper right (b) is TritC. Bottom left (c) is Cy-5 and bottom right (d) is “Hope.” Identical section of slide. Note the positive signal in this “No-probe” control. As in Additional file 10, without probes although there is a slight difference in intensity, “positive” signal is seen with both sets of filters. [file 13104_2018_3601_MOESM11_ESM.tif]

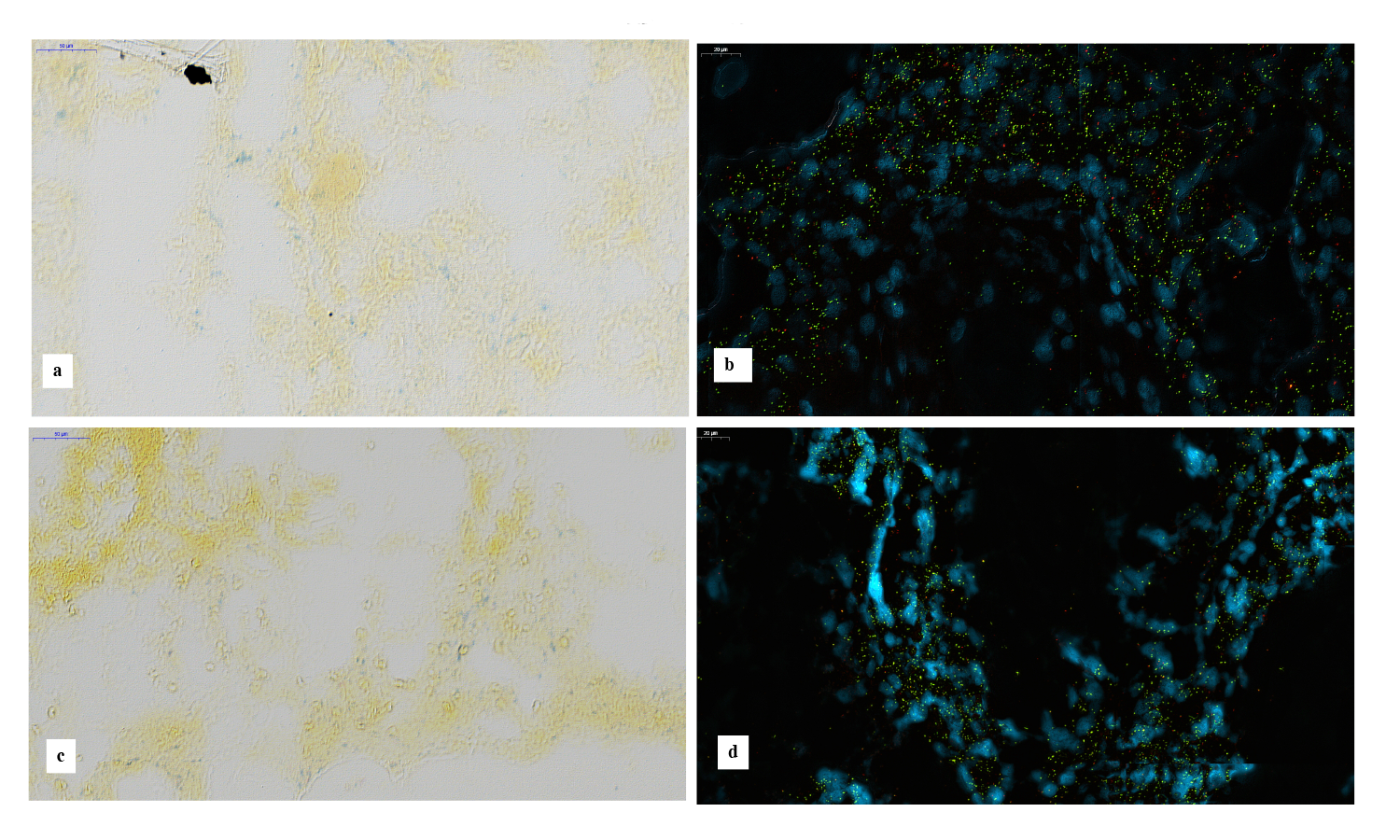

Supplement: Supplementary file 12 — Additional file 12. A comparison, using an alternative imager (HisTech®. See “Main text” section for details.) With (a & b) & without probes (c & d). Brightfield are a & c. Composite fluorescent of DAPI, Cy-5 & Texas-Red are b & d. “Positive” fluorescent signal is seen for both CY5 and Texas-Red in both b & d. Note difference in magnification between Brightfield and Fluorescent images. [file 13104_2018_3601_MOESM12_ESM.tif]

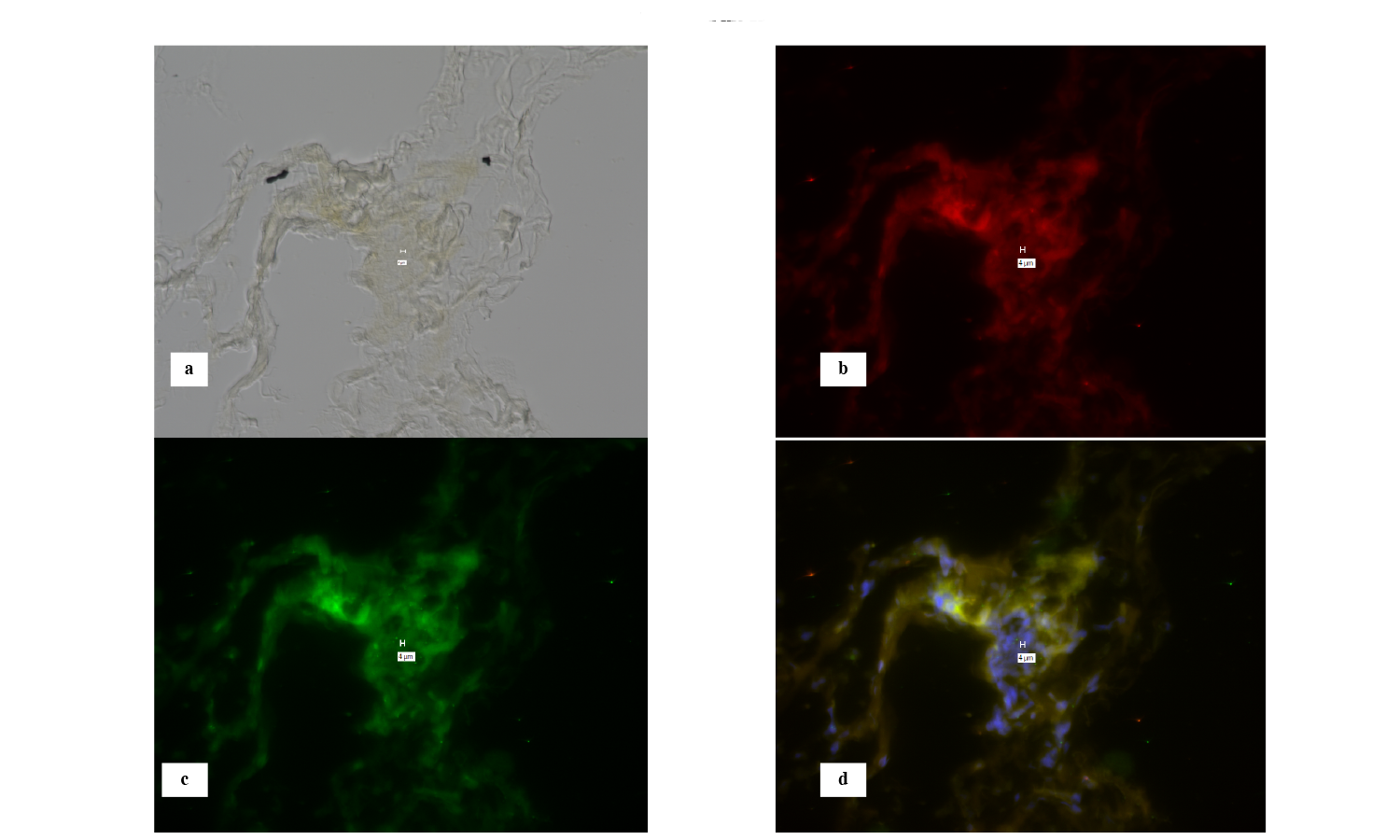

Supplement: Supplementary file 13 — Additional file 13. A “No-probe” control on a specimen from Ames Iowa visualized with Bright Field (Additional file 13 a) Texas Red (Additional file 13 b) and Cy-5 (Additional file 13 c) Composite of DAPI, Texas Red & Cy5 (Additional file 13 d.) Note the positive signal in this “No-probe” control. [file 13104_2018_3601_MOESM13_ESM.tif]

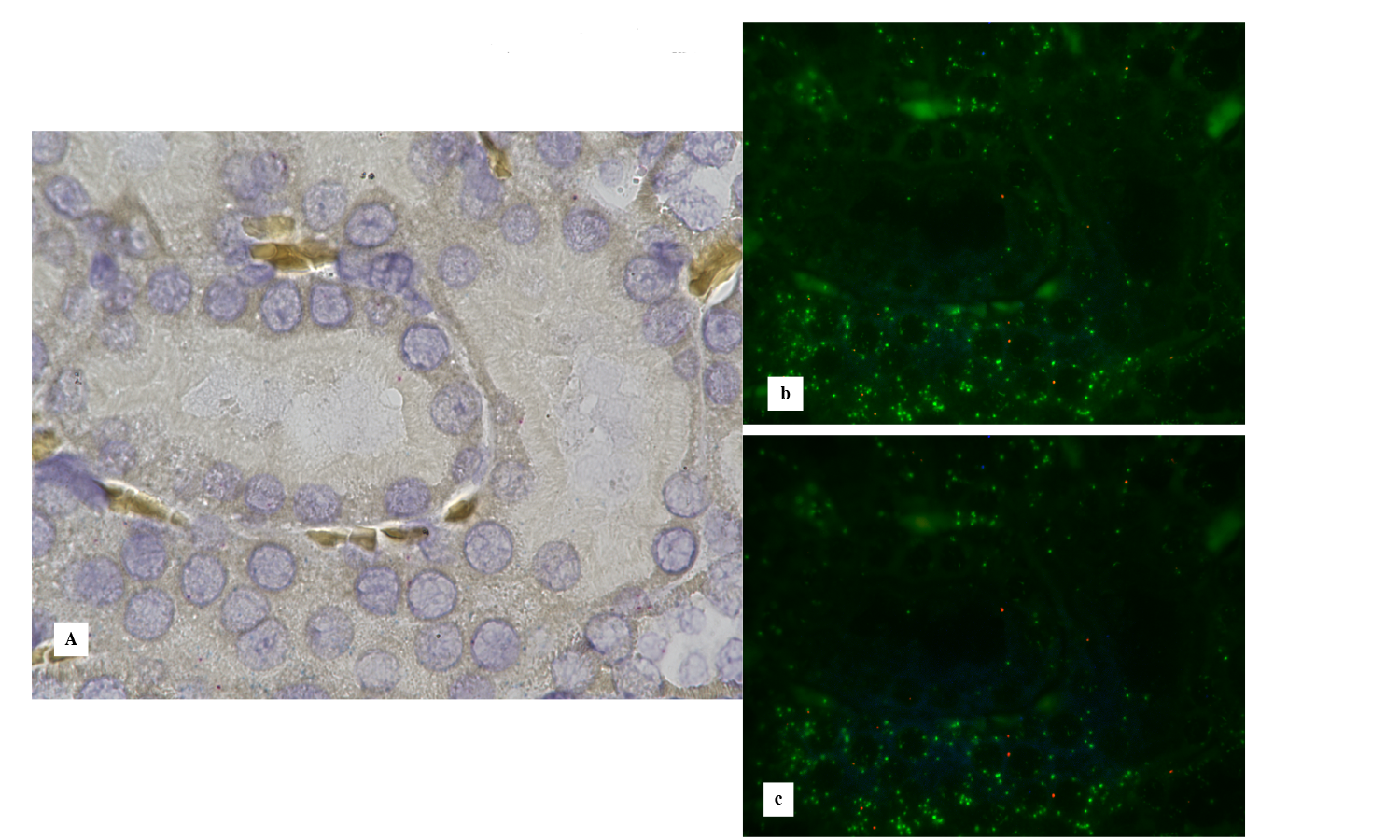

Supplement: Supplementary file 14 — Additional file 14. Affymetrix supplied Rat Kidney “No-probe” control slide. “a” = Bright field x100. “b” = Composite of DAPI, Texas Red & Cy-5. “c” = Composite of DAPI, TritC and “Hope”. Note the positive signal in the “No-probe” control. [file 13104_2018_3601_MOESM14_ESM.tif]
